# Editorial: Molecules Balancing Immunological Surveillance against Cancer and Autoimmune Diseases

**DOI:** 10.3389/fonc.2016.00086

**Published:** 2016-04-12

**Authors:** Amir Sharabi

**Affiliations:** ^1^Department of Clinical Immunology, Sackler Faculty of Medicine, Tel-Aviv University, Tel-Aviv, Israel; ^2^Department of Rheumatology, Tel-Aviv Sourasky Medical Center, Tel-Aviv, Israel

**Keywords:** editorial, innate immune system, adaptive immune system, pathologies, cancer, autoimmune diseases

**The Editorial on the Research Topic**

**Molecules Balancing Immunological Surveillance against Cancer and Autoimmune Diseases**

The innate and the adaptive immune systems work in concert to deal with pathologies introduced by internal and external factors (e.g., viruses, bacteria, and fungi) – some of which may be associated with the development of autoimmune and cancerous cells. Mechanistically, there are internal factors that are responsible for the hypo- and hyperfunction of immune tolerance apparatus. Consequently, it could lead to the development of autoimmune diseases and malignancies, respectively. In the last decade, the study of cancer and autoimmunity suggested that these two entities might reflect two opposed extreme end-points of an immune response spectrum. Accordingly, the overexpression of certain molecules could result in malignancies, whereas deficient expression of the same molecules could lead to the development of autoimmune diseases.

Two journals, Frontiers in Immunology and Frontiers in Oncology, participated in this special issue in an attempt to gain the prospective of professionals in both fields of autoimmunity and cancer, hoping to identify molecules and factors that possess dual roles with opposing effects on autoimmune diseases and cancer.

E3 ligase Cbl-b, as reviewed by Lutz-Nicoladoni et al., is central in the balance of activating and inhibitory inputs to immune cells. Specifically, Cbl-b negatively regulates activation signals through antigen or pattern recognition receptors and costimulatory molecules. Although *cblb*-deficient immune cells display lower activation thresholds and *cblb* knockout mice spontaneously develop autoimmunity and are highly susceptible to experimental autoimmunity, the increased activation potential of *cblb*-deficient cells renders them more potent to fight against malignancies or infections.

In the study of multiple sclerosis (MS), Kaushansky and Ben-Nun, report that the allele DQB1*06:02 shown previously to be a disease-modifying allele, also plays a role as a disease-predisposing allele. They review a novel “humanized” model of MS-like disease, in which autoimmunity against myelin may be driven by a pathogenic HLA-DQ expression. This model also supports an additional target autoantigen, e.g., myelin-associated oligodendrocytic basic protein (MOBP) in the pathogenesis of MS.

Haque et al. summarize the utilization of regulatory T cells (Treg cells) in treating rheumatoid arthritis (RA). Because Foxp3 can be polyubiquitinated, they show that the expression of Foxp3 in T cells can be facilitated through Foxp3 ubiquitination. Ubiquitin-specific-processing protease 7 (USP7) is an active enzyme in Treg cells, which may be associated with Foxp3, and leads to its deubiquitination. By contrast, diminished function of USP7 results in a decreased function of Treg cells.

In RA, Treg cells do not possess normal immune suppressive activity, although they can be found in the joints of the patients. It is explained, in part, by the elevated levels of TNF-α within the inflamed synovium and the joint fluid, which may lead to Foxp3 phosphorylation, thereby interfering with the suppressive capacities of Treg cells. Recent studies demonstrated that induced pluripotent stem cell (iPSC) can be reprogramed to constitute a source for functional CD4^+^ Treg cells or CD8^+^ cytotoxic T lymphocytes. The adoptive transfer of the latter cells may be used for immunotherapy of autoimmune arthritis and cancers. Hence, generation of antigen-specific Foxp3^+^ Treg cells from stem cells, such as iPSCs, may open a new area in Treg cell-based immunotherapy in transplantation and autoimmune disorders.

Manjili et al. describes the evolution of myeloid regulatory cells (Mregs). These cells suppress (through direct and indirect mechanisms) the host’s anti-tumor immune responses in favor of the tumor. By contrast, the ability of Mregs to limit and modulate Th1 responses or support a skewed Th2 immunity could be beneficial during parasitic and helminth infections. In addition, the immunoregulatory functions of Mregs on Th17 differentiation and inflammation were demonstrated in experimental autoimmune encephalomyelitis (EAE). In this model, treatment with gemcitabine that depleted Mregs led to a reduction in Th17 cells, IL-17A, and IL-1β in the lymphoid tissues and spinal cord and to a substantial decrease in the severity of EAE. In addition, Mregs were shown to induce Th17 differentiation in tumor-bearing mice and in patients with ovarian cancer. Also, Mregs’ levels correlated the levels of Th17 cells or IL-17 production in patients with gastrointestinal cancer. Because of the association observed between inflammation, tumorigenesis, and the progression of autoimmune diseases, these proinflammatory cells may represent a pivotal pathogenic factor in the spectrum of cancer and autoimmunity.

## Author Contributions

The author confirms being the sole contributor of this work and approved it for publication.

## Conflict of Interest Statement

The author declares that the research was conducted in the absence of any commercial or financial relationships that could be construed as a potential conflict of interest.

